# The clinical and prognostic significance of FOXN3 downregulation in acute myeloid leukaemia

**DOI:** 10.1111/ijlh.13162

**Published:** 2020-02-20

**Authors:** Jinjing Zhang, Yue Wang, Wenbin Mo, Rui Zhang, Yan Li

**Affiliations:** ^1^ Department of Hematology The First Affiliated Hospital of China Medical University Shenyang China

**Keywords:** acute myeloid leukaemia, chemotherapy response, diagnosis, FOXN3, prognosis

## Abstract

**Introduction:**

The expression of forkhead box N3 (FOXN3), also known as checkpoint suppressor 1 (CHES1), is reduced in many types of tumours. However, the clinical significance of FOXN3 and its potential role in acute myeloid leukaemia (AML) remain largely unknown.

**Methods:**

A total of 117 de novo AML patients newly diagnosed between December 2015 and January 2018 were included in this study. The expression of FOXN3 and its clinical significance were analysed in these AML patients.

**Results:**

The expression of FOXN3 was significantly downregulated in AML. In addition, lower FOXN3 expression was associated with older age and higher white blood cell counts. Moreover, a close correlation was observed between lower FOXN3 expression and a lower complete remission (CR) rate and shorter overall survival (OS), which was further analysed by multivariate analysis.

**Conclusion:**

These data suggest that FOXN3 is a novel biomarker in AML and that lower FOXN3 expression predicts poor chemotherapy response and prognosis in AML.

## 1 INTRODUCTION

Acute myeloid leukaemia (AML), the most common type of adult leukaemia, is characterized by out‐of‐control proliferation, the inhibition of differentiation, the apoptotic blockage of leucocytes and reduction in normal haematopoietic cells.^1^, ^2^ With the development of cytogenetic and molecular biology, AML could be diagnosed and treated at the genomic level with a better therapeutic effect. However, 60%‐80% of AML patients could not be cured due to disease resistance and recurrence.^3^, ^4^, ^5^, ^6^, ^7^, ^8^ Recent studies that focused on abnormal transcription demonstrate the key role of transcription regulators in leukaemogenesis and suggest a potential therapeutic strategy for AML.^5^ Therefore, the identification of novel abnormal transcription factors and their functions in AML will provide new clues on the pathogenesis and treatment of AML.

The transcription factor forkhead box N3 (FOXN3), as a member of the forkhead box N superfamily, participates in several biological processes, including the cell cycle, cell differentiation, epithelial‐mesenchymal transition, gene transcription and glucose metabolism.^9^, ^10^, ^11^, ^12^, ^13^ Previous studies have shown that the downregulated expression of FOXN3 is observed in various malignancies, such as hepatocellular carcinoma (HCC), colon cancer, ERα‐positive breast cancer, Hodgkin lymphoma, head and neck cancer, lung cancer, adult glioblastoma multiforme, T cell acute lymphoblastic leukaemia (ALL) and osteosarcoma,^9^, ^10^, ^12^, ^14^, ^15^, ^16^, ^17^, ^18^, ^19^ and the FOXN3 expression level is associated with the prognosis of some cancers.^9^, ^12^, ^17^, ^19^

Although lower FOXN3 expression in adult AML was found in our previous study,^20^, ^21^ its clinical and prognostic significance in AML remains unknown. Our study investigated the expression profile of FOXN3 in the bone marrow (BM) of adult AML patients and analysed its clinical significance.

## 2 MATERIALS AND METHODS

### 2.1 Patients

The present study enrolled 117 newly diagnosed AML patients between December 2015 and January 2018 and 25 healthy donors at the First Affiliated Hospital of China Medical University in China. The diagnosis was made according to the French‐American‐British (FAB) Cooperative Group criteria.^22^ The BM samples were analysed using flow cytometric immunophenotyping, conventional chromosome banding or targeted analyses [reverse transcriptase quantitative polymerase chain reaction (RT‐qPCR) and/or fluorescence in situ hybridization (FISH)] and next‐generation sequencing (NGS) with a Genoptix panel including 21 AML‐associated genes, such as ASXL1, CEBPA, DNMT3A, FLT3, GATA2, IDH1, IDH2, KIT, KRAS, MLL, NPM1, NRAS, PHF6, RUNX1, TET2, TP53, WT1, SF3B1, SRSF2, U2AF1 and ETV6. Acute promyelocytic leukaemia (APL), the M3 subtype of AML which was characterized with *t*(15;17)/PML‐RARa fusion gene, was excluded from this study. This study was approved by the Ethics Committee of the First Affiliated Hospital of China Medical University.

### 2.2 Therapy and follow‐up

The follow‐up information of 96 AML patients was available. The treatment followed protocol described as our previous study and response assessment was based on Chinese expert consensus on the treatment of AML (2011).^20^, ^23^ A total of 12 patients received haematopoietic stem cell transplantation (HSCT) in the CR phase. No further therapy was applied to patients remaining in remission after postremission therapy. The follow‐up time for the patients was calculated from the time of randomization for induction therapy to November 2018 unless death occurred. BM samples from patients with AML at the time points of diagnosis, CR were included in the analysis.

### 2.3 RNA isolation and RT‐qPCR

Ficoll‐Paque™PLUS (GE Healthcare) was used to extract mononuclear cells from BM. Total RNA was isolated utilizing TRIzol reagent (Invitrogen), and cDNA was prepared from 1 µg of RNA using the PrimeScript™RT Reagent Kit with gDNA Eraser (TaKaRa). For the detection of FOXN3 expression levels in the BM of patients and normal controls, real‐time quantitative PCR (RT‐qPCR) was conducted using a TaqMan Gene Expression Assay on an ABI 7500 Real‐Time PCR system (Applied Biosystems) as previously described,^20^ and ABL was used as a control gene. The primers and TaqMan‐based probes were as follows: FOXN3 forward 5′‐TGCCAATCACTCCCATTGGG‐3′, reverse 5′‐CCGCATCCGGCAGCTGG‐3′ and probe Fam‐TGCCATTCCTCATGGCCGCTGTCA‐Tam; and ABL forward 5′‐TGGAGATAACACTCTAAGCATAACTAAAGGT‐3′, reverse 5′‐GATGTAGTTGCTTGGGACCCA‐3′ and probe Fam‐CCATTTTTGGTTTGGGCTTCACACCATT‐Tam. For the detection of PIM2 and E2F5 expression levels in the BM of patients, RT‐qPCR was conducted using SYBR Green technology as previously described.^21^ The primers used are shown in Table S1.

### 2.4 Immunocytochemistry

The cytospin smears of BM cells from AML and normal control samples were fixed in paraformaldehyde (4%, 5 minutes). The specimen was then incubated with a peroxidase‐blocking enzyme and normal goat serum (10 minutes each), followed by incubation with rabbit antihuman FOXN3 protein antibodies (HPA059209, SIGMA) at 37°C for 30 minutes. Biotin‐labelled goat antirabbit IgG was used as a secondary antibody, and the protein was detected using the streptavidin‐peroxidase (SP) complex developed with DAB according to the manufacturer's instructions. Subsequently, the specimens were counter‐stained with haematoxylin. Finally, the reactivity of the antibody was made visible with the Vector brown SP substrate.

### 2.5 Gene expression data set

Forkhead box N3 expression was compared between haematopoietic stem cells (HSCs) and AML from the Bloodpool data set (probe number: 222494) using the online BloodSpot database (www.bloodspot.eu).^24^

### 2.6 Statistical analysis

Statistical analysis was performed by GraphPad Prism 7.0a software and SPSS 15.1 software. Differences between groups were compared using the Mann‐Whitney test or one‐way analysis of variance (ANOVA) among multiple groups. Pearson chi‐square analysis/Fisher's exact test was conducted to compare the differences of categorical variables. Survival analysis was used to analyse the impact of FOXN3 on relapse‐free survival (RFS) and overall survival (OS), and the differences were compared by a log‐rank test. Univariate and multivariate analyses were performed using the Cox promotional hazards regression model.

## 3 RESULTS

### 3.1 FOXN3 expression was abnormally downregulated in AML

**Figure 1:**
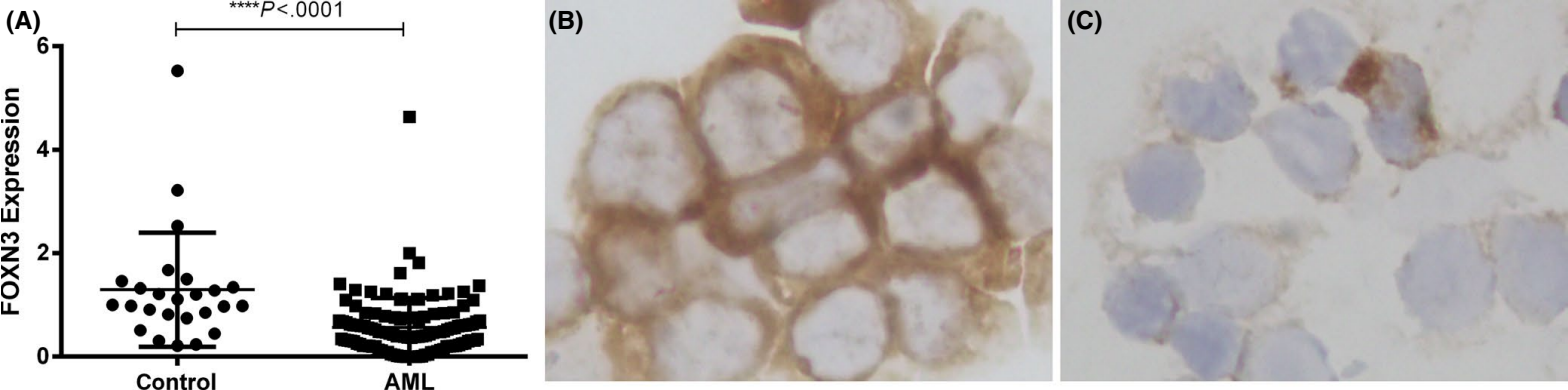
FOXN3 expression is significantly downregulated in AML. A, Expression levels of FOXN3 mRNA were detected in AML patients and controls by RT‐qPCR. ****P < .0001. B and C, Representative images showing decreased expression levels of FOXN3 protein in control (B) and AML patients (C) using immunocytochemical staining [Colour figure can be viewed at wileyonlinelibrary.com]

Forkhead box N3 mRNA expression was detected in a total of 117 AML patients and 25 controls by RT‐qPCR. As shown in Figure 1, the FOXN3 mRNA levels in AML patients (median: 0.468, range: 0.000‐4.640) were significantly downregulated compared with those in the controls (median: 1.000, range: 0.214‐5.525) (*P* < .0001, Figure 1A). Moreover, the lower expression of FOXN3 protein was confirmed in 15 AML patients with decreased levels of FOXN3 mRNA by SP immunocytochemical staining (Figure 1B,C). This downregulated expression of FOXN3 in AML was validated by analysing the online Bloodpool data set (www.bloodspot.eu), revealing that FOXN3 expression was significantly lower in AML than in CD34+ HSCs.

### 3.2 Lower FOXN3 expression correlated with older age and higher WBC

The 117 AML patients were divided into two groups according to whether their FOXN3 expression levels were below (lower expression group) or above (higher expression group) the median level of FOXN3 expression. The comparison of clinical features between the two groups showed that a lower expression of FOXN3 was correlated with older age and higher white blood cell counts (*P* = .033 and .032, respectively, Table 1). There were no significant differences observed in haemoglobin, platelet counts, BM blasts, FAB subtypes, cytogenetic subgroups and prognostic risk stratification^25^ (Table 1).

**Table 1:** Comparison of clinical characteristics between AML patients with lower and higher FOXN3 expression

Parameters	Lower FOXN3 expression, n = 58	Higher FOXN3 expression, n = 59	*P*
Sex, male/female	35/23	31/28	.395
Median age, years (range)	52 (16 ~ 78)	52 (14 ~ 80)	.033
<60	37 (45.53%)	48 (56.47%)	
≥60	21 (65.63%)	11 (34.38%)	
Median WBC, ×10 ~ 9/L (range)	28.65 (0.87 ~ 231.63)	13.05 (0.89 ~ 368.23)	.032
<30	28 (41.18%)	40 (58.82%)	
≥30	30 (61.22%)	19 (38.78%)	
Median HB, g/L (range)	80 (43 ~ 133)	82 (20 ~ 144)	.646
<80	29 (51.79%)	27 (48.21%)	
≥80	29 (47.54%)	32 (52.46%)	
Median PLT, ×10 ~ 9/L (range)	49.5 (4 ~ 645)	32 (3 ~ 365)	.078
<50	29 (42.65%)	39 (57.35%)	
≥50	29 (59.18%)	20 (40.82%)	
BM blast, % (range)	68 (12 ~ 97.6)	64.8 (0 ~ 93.2)	.150
<80	35 (44.87%)	43 (55.13%)	
≥80	23 (58.97%)	16 (41.03%)	
FAB, n			
M0	0	1	.993
M1	2	2	.623
M2	17	20	.594
M4	1	2	.533
M5	38	32	.213
M6	0	2	.496
Cytogenetic risk, n			
favourable risk	8	14	.335
Intermediate risk	28	27	.785
poor risk	9	5	.241
No data	13	13	.961

Abbreviations: AML, acute myeloid leukaemia; BM, bone marrow; CR, complete remission; FAB, French‐American‐British; favourable risk: t(8;21), inv(16) or t(16;16); HB, haemoglobin; Intermediate risk: normal cytogenetics, other nondefined; PLT, platelet; poor risk: complex, −7, 11q23‐non t(9;11), t(9;22); PR, partial remission; WBC, white blood cells.

### 3.3 Association of FOXN3 expression with cytogenetic and molecular abnormalities

**Figure 2:**
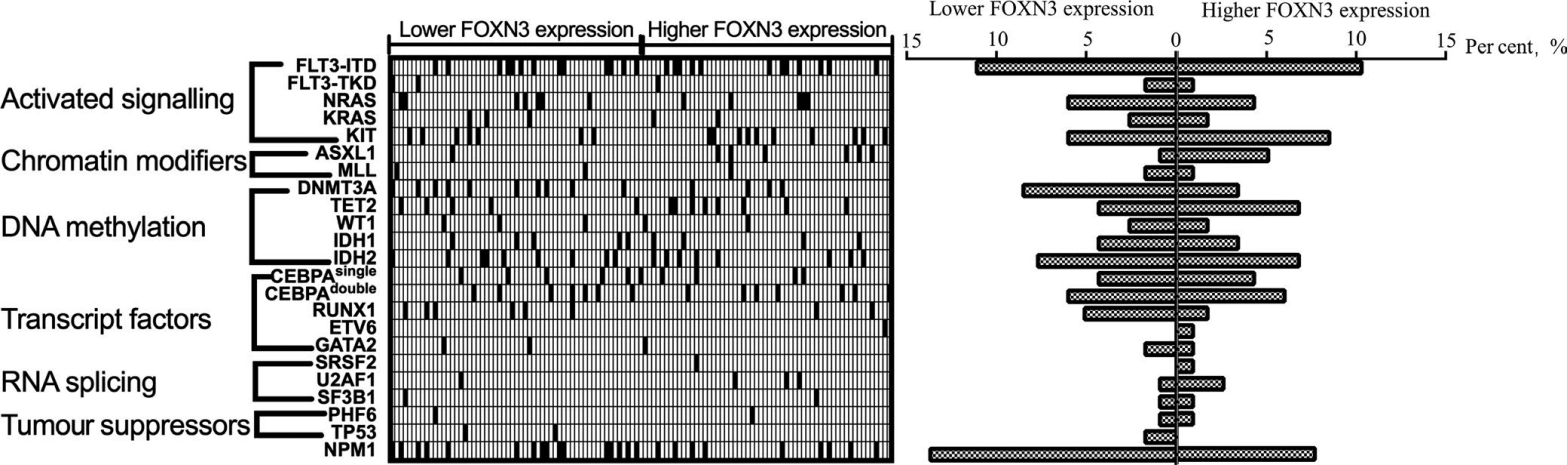
Relationships between FOXN3 expression levels and other common gene mutations in 117 patients with AML

A total of 202 gene mutations were demonstrated in the 117 AML patients. The most frequently mutated genes in our study were FLT3 (28/117, 23.9%), IDH1/2 (26/117, 22.2%), NPM1 (25/117, 21.4%) and CEBPA^double/single^ (24/117, 20.5%), followed by KIT (17/117, 14.5%), N/K‐RAS (17/117, 14.5%), DNMT3A (14/117, 12.0%) and TET2 (13/117, 11.1%). The mutations of 21 genes were classified into seven types according to their contribution to leukaemogenesis: genes inducing activated signalling (FLT3‐ITD/TKD, N/KRAS and KIT), chromatin modifiers (ASXL1 and MLL), DNA methylation (DNMT3A, TET2, WT1 and IDH1/2), transcription factors (CEBPA, RUNX1, ETV6 and GATA2), RNA splicing (SRSF2, SF3B1 and U2AF1), tumour suppressors (PHF6 and TP53) and NPM1 (Figure 2). No significant difference in gene mutations was observed between the lower FOXN3 expression group and the higher FOXN3 expression group. In addition, patients with lower FOXN3 expression showed a higher tendency of DNMT3A and NPM1 mutations and lower incidences of ASXL1 mutations (*P* = .145, 0.104 and 0.125, respectively). TP53 mutations (n = 2) occurred in only the lower FOXN3 expression group.

### 3.4 Lower FOXN3 expression is associated with poor chemotherapy response and shorter survival in AML

**Figure 3:**
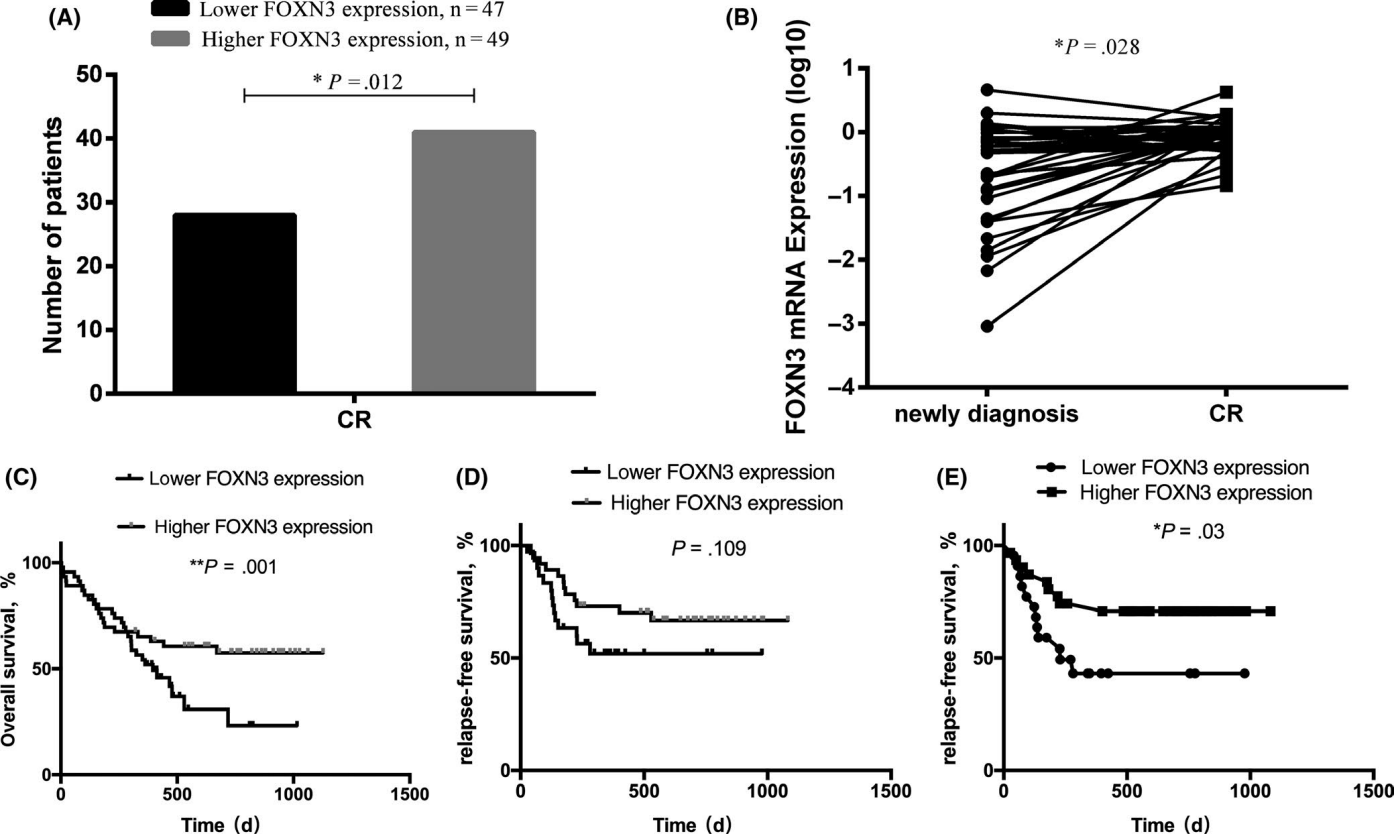
Lower FOXN3 expression is associated with poor chemotherapy response and shorter OS in AML. A, The comparison of therapeutic effects between AML patients with lower FOXN3 expression and those with higher FOXN3 expression. B, FOXN3 expression was significantly higher in the CR phase compared to that at the newly diagnosed time point (*P = .028). C and D, The impact of FOXN3 on OS and RFS in AML patients. E, The RFS of the higher FOXN3 expression group was significantly longer than that of the lower FOXN3 expression group when older patients (>60 y) were excluded

Follow‐up data were collected from 96 AML patients, and the clinical information is summarized in Table S2. A total of 69 (72%) patients achieved CR after induction chemotherapy and 27 (28%) patients experienced induction chemotherapy failure. AML patients with lower FOXN3 expression showed a significantly lower CR rate than those with higher FOXN3 expression group (*P* = .012, Figure 3A). To investigate the changes in FOXN3 expression regarding different disease statuses in AML, we further detected expression of FOXN3 in 34 paired patients who achieved CR. The data suggested that FOXN3 expression was significantly higher in the CR phase (median: 0.949, range: 0.146‐4.230) than at the newly diagnosed time point (median: 0.219, range: 0.001‐4.640) (*P = *.028, Figure 3B). Among these 34 patients, 16 were allocated to the lower FOXN3 expression group and the other 18 to the higher FOXN3 expression group. In the lower FOXN3 expression group, all 16 patients showed significantly higher FOXN3 mRNA expression at CR than when newly diagnosed (*P* = .0007, Figure S1A), whereas no significant difference was observed in the higher FOXN3 expression group (Figure S1B).

Survival analysis was performed to compare RFS and OS between the FOXN3 lower expression and higher expression groups. The results indicated that patients with lower FOXN3 expression presented a significantly shorter OS time than those with higher FOXN3 expression (Figure 3C). Although it was lack of significant difference in RFS between the two groups (Figure 3D), the RFS of the higher FOXN3 expression group was significantly longer than that of the lower FOXN3 expression group when older patients were excluded (Figure 3E). Univariate and multivariate analyses were further performed to reveal the prognostic significance of FOXN3 expression in AML according to ELN recommendations and previous studies 6, 26, 27 (Table 2). The multivariate analysis showed that the expression of FOXN3 was an independent prognostic factor correlated with OS (HR = 0.269, *P* = .003).

**Table 2:** Univariate and multivariate analysis of prognostic factors for overall survival in AML patient

	Univariate analysis		Multivariate analysis	
	Hazard ratio(95% CI)	*P*	Hazard ratio(95% CI)	*P*
Age	1.413 (0.675‐2.956)	.359	1.522 (0.660‐3.511)	.324
WBC	2.349 (1.200‐4.599)	.013	3.729 (1.540‐9.028)	.004
Sex	1.438 (0.741‐2.792)	.283	1.500 (0.599‐3.753)	.386
BM blast	1.765 (0.884‐3.522)	.107	0.476 (0.205‐1.106)	.084
FOXN3	0.413 (0.204‐0.837)	.014	0.269 (0.113‐0.641)	.003
Risk stratification	2.572 (1.480‐4.470)	.001	3.884 (1.692‐8.915)	.001
FLT3‐ITD	2.927 (1.469‐5.836)	.002	0.634 (0.196‐2.047)	.446
KIT	0.236 (0.057‐0.983)	.047	0.642 (0.137‐3.007)	.496
ASXL1	2.266 (0.692‐7.423)	.177	12.927 (2.757‐60.608)	.001
CEBPA^double^	0.602 (0.184‐1.969)	.401	1.029 (0.217‐4.868)	.972
RUNX1	1.871 (0.444‐7.878)	.393	1.412 (0.235‐8.476)	.706
NPM1	4.547 (2.134‐9.687)	.000	7.043 (2.579‐19.235)	.000

Note

Variables were composed of age (<60 vs ≥60 y), WBC (<30 vs ≥30*10 ~ 9/L), Sex (male vs female), BM blasts (<80% vs ≥80%), FOXN3 expression (lower vs higher), Risk stratification (favourable vs others) and gene mutation (wild type vs mutant).

Abbreviation: CI, confidence interval.

### 3.5 Association of FOXN3 expression with PIM2 and E2F5 expression

To investigate the target genes of FOXN3 as a transcriptional suppressor in AML, the mRNA expression of PIM2 and E2F5 in the BM of 32 patients was detected by RT‐qPCR. Unfortunately, there was no negative correlation between FOXN3 and PIM2 or E2F5 (Figure S2).

## 4 DISCUSSION

Forkhead box N3 belongs to the FOXN gene family and was first discovered as a suppressor of DNA damage‐activated checkpoint mutations in yeast.^28^ In recent years, studies on FOXN3 have suggested that it may play dual roles in different tumours. Acting as a tumour suppressor gene, FOXN3 is reduced in several types of tumours, such as HCC, colon cancer and osteosarcoma,^9^, ^10^, ^19^ but it is upregulated in ovarian cancer and breast cancer, where it acts as an oncogene.^11^, ^29^ One explanation for the diverse effects of FOXN3 may be due to the specific cellular and tissue environment.^30^, ^31^ In our previous studies, the forced expression of FOXN3 inhibited cell proliferation, the induction of apoptosis, and cell cycle arrest.^21^ These results indicated that FOXN3 may participate in the malignant transformation of leukaemia cells. In this study, the significant downregulation of FOXN3 was validated, and FOXN3 was also indicated to be an independent prognostic marker of AML. These findings affirmed the tumour suppressive role of FOXN3 in AML.

As a tumour suppressor, the impact of FOXN3 on prognosis has been demonstrated in solid tumours. Patients with high FOXN3 expression had longer OS and RFS times than those with low FOXN3 expression in HCC, osteosarcoma and breast cancer patients.^9^, ^12^, ^19^ The results of this study suggested that downregulation of FOXN3 correlates with poor OS and RFS of non‐APL. The level of FOXN3 is not only an independent prognostic factor but also serves as a biomarker of treatment response in non‐APL. However, it seems that the prognostic effect of FOXN3 expression was inconsistent with the public data from the Cancer Genome Atlas (TCGA) (http://www.cbioportal.org)^7^, ^32^ and Beat AML database (http://www.vizome.org)^33^ even if APLs were removed (Figure S3). A significant difference in RFS between the two groups with different levels of FOXN3 expression was observed when older patients were excluded, suggesting that treatment bias, such as the dose‐adjusted regimen for aged patients, should be taken into consideration. Therefore, prospective clinical trials with more cases are needed to validate the prognostic value of FOXN3 in AML.

In this study, the abnormal expression of FOXN3 showed a tendency but not significant difference on mutations of NPM1, DNMT3A, TP53 and ASXL1, suggesting the potential correlation of FOXN3 with molecular aberrations. By searching the publicly available data containing a larger cohort of patients,^33^ the correlation of lower FOXN3 expression with higher incidence of NPM1 mutation could be confirmed, whereas there was no significant distinction on mutations of DNMT3A, TP53 and ASXL1 between AML with different FOXN3 levels (Figure S4).

As a transcription regulator, FOXN3 has been reported to inhibit the expression of some tumour oncogenes, such as PIM2 and E2F5.^9^, ^34^ However, the negative correlation between FOXN3 and PIM2 or E2F5 was not validated in this study. Considering that FOXN3 expression in AML is low, quantification of PIM2 and E2F5 transcript levels in transformed AML cell lines with overexpression of FOXN3 is needed to further clarify the regulation of PIM2 and E2F5 by FOXN3 in AML.

In summary, FOXN3 was downregulated in AML patients and associated with older age and higher white blood cell counts, and the expression of FOXN3 was higher in the CR phase. Patients with lower FOXN3 expression had a lower CR rate and shorter OS. Our study suggested that FOXN3 may be a novel potential biomarker of AML that could predict poor chemotherapy response and prognosis in AML.

## CONFLICT OF INTEREST

The authors have no competing interest.

## AUTHOR CONTRIBUTION

JJ Zhang, R Zhang and Y Li contributed to the conception and design of the work, data collection, data analysis and wrote the manuscript draft. JJ Zhang and Y Wang participated in the experiments. WB Mo assisted in completing the statistical analysis and drafted the figures. R Zhang and Y Li are co‐corresponding authors of this article and responsible for this study. All authors read and approved the final manuscript.
