# Supplementary material for: The clinical and prognostic significance of FOXN3 downregulation in acute myeloid leukaemia
Source: Int J Lab Hematol. 2020 Feb 20;42(3):270–6. doi: 10.1111/ijlh.13162 (PMC7317382; doi:10.1111/ijlh.13162)

Table S1

Gene Name

Sequence (5’-3’)

*PIM2*

F: GTGGGGACATTCCCTTTGAGAG

R: GGATTAGGGCACAGCAGTCTGG

F:TCTTCAGCAGGATCTATTAGTGG

R:TGTAGTCATCTGCCGGGGTA

F: CACTGTGCCCATCTACGAGG

*E2F5*

*β-actin*

(control gene) R: TAATGTCACGCACGATTTCC

*FOXN3*

F：TGCCAATCAACTCCCATTGGG

R：CCGCATCCGGCAGCTGG


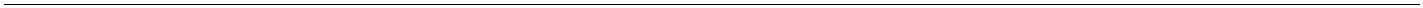

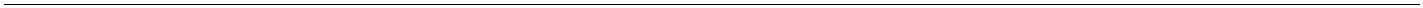

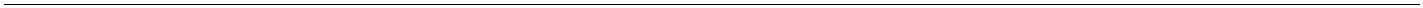


Table S2

Parameters

Lower FOXN3

expression, n=47

27/20

Higher FOXN3

expression, n=49

25/24

*P*

Sex, male/female

age, years

<60

0.527

0.413

33

14

38

11

≥60

WBC,×10~9/L

<30

≥30

HB, g/L

<80

0.227

0.426

25

22

32

17

22

25

19

30

≥80

PLT,×10~9/L

<50

≥50

BM blast,%

<80

≥80

0.015

0.139

20

27

33

16

30

17

38

11

FAB, n

M1

M2

M4

M5

2

14

1

30

0

2

19

2

25

1

0.966

0.996

0.582

0.204

0.330

M6

Cytogenetic risk, n

favorable-risk

Intermediate-risk

poor-risk

No data

Induction

response, n

CR

5

6

21

13

9

0.805

0.375

0.562

0.543

16

15

11

28

14

5

41

6

2

0.009

0.034

0.263

PR+ refractory

Induction death

AML, acute myeloid leukemia; WBC, white blood cells; HB, hemoglobin; PLT,

platelet; BM, bone marrow; FAB, French-American-British; favorable-risk: t(8;21),

inv(16) or t(16;16); Intermediate-risk: normal cytogenetics, other non-defined; poor-

risk: complex, -7, 11q23-non t(9;11), t(9;22); CR, complete remission; PR, partial

remission.


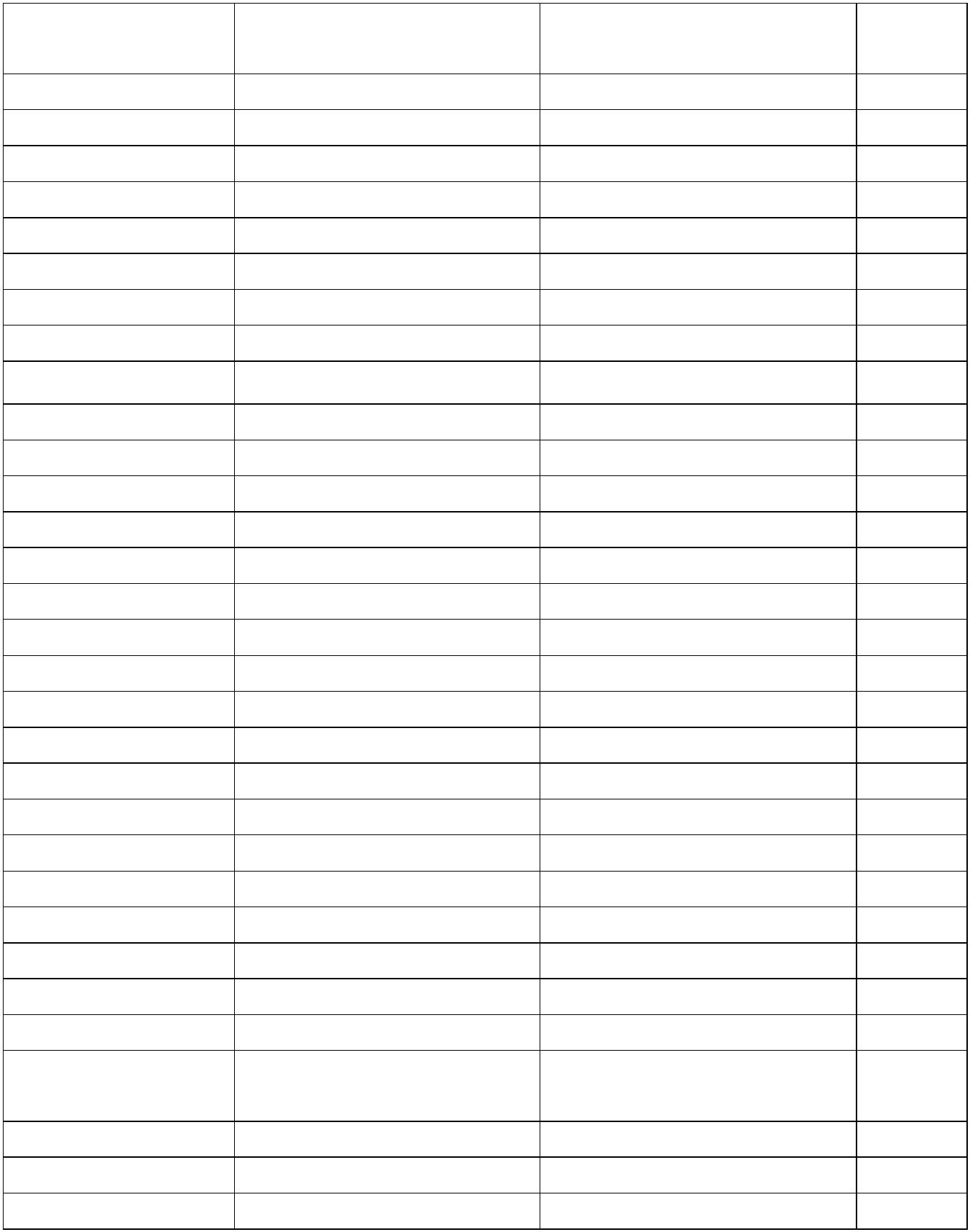


**A**

**B**

**1**

**0**

**1.0**

****P=0.0007*

*P*=0.609

**0.5**

**0.0**

**-1**

**-2**

**-3**

**-4**

**-0.5**

**newly diagnosis**

**CR**

**newly diagnosis**

**CR**

Figure S1: The dynamic change of FOXN3 mRNA expression at CR

and newly diagnosis phase in lower FOXN3 expression group(A, n=16) and

higher FOXN3 expression group(B, n=18).


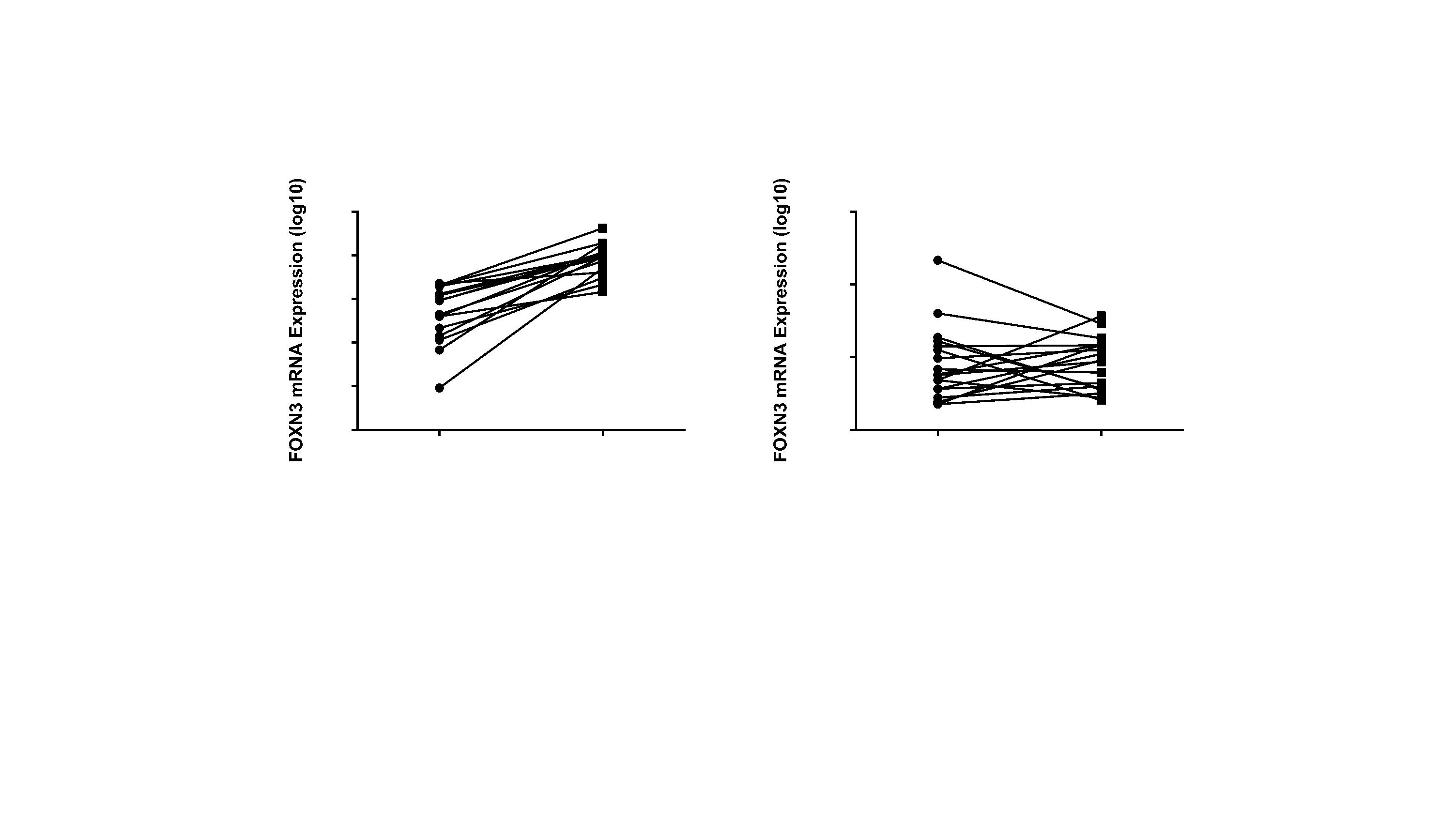


**B**

**A**

Figure S2: The correlation between FOXN3 and PIM2(A) or E2F5(B) (n=32).


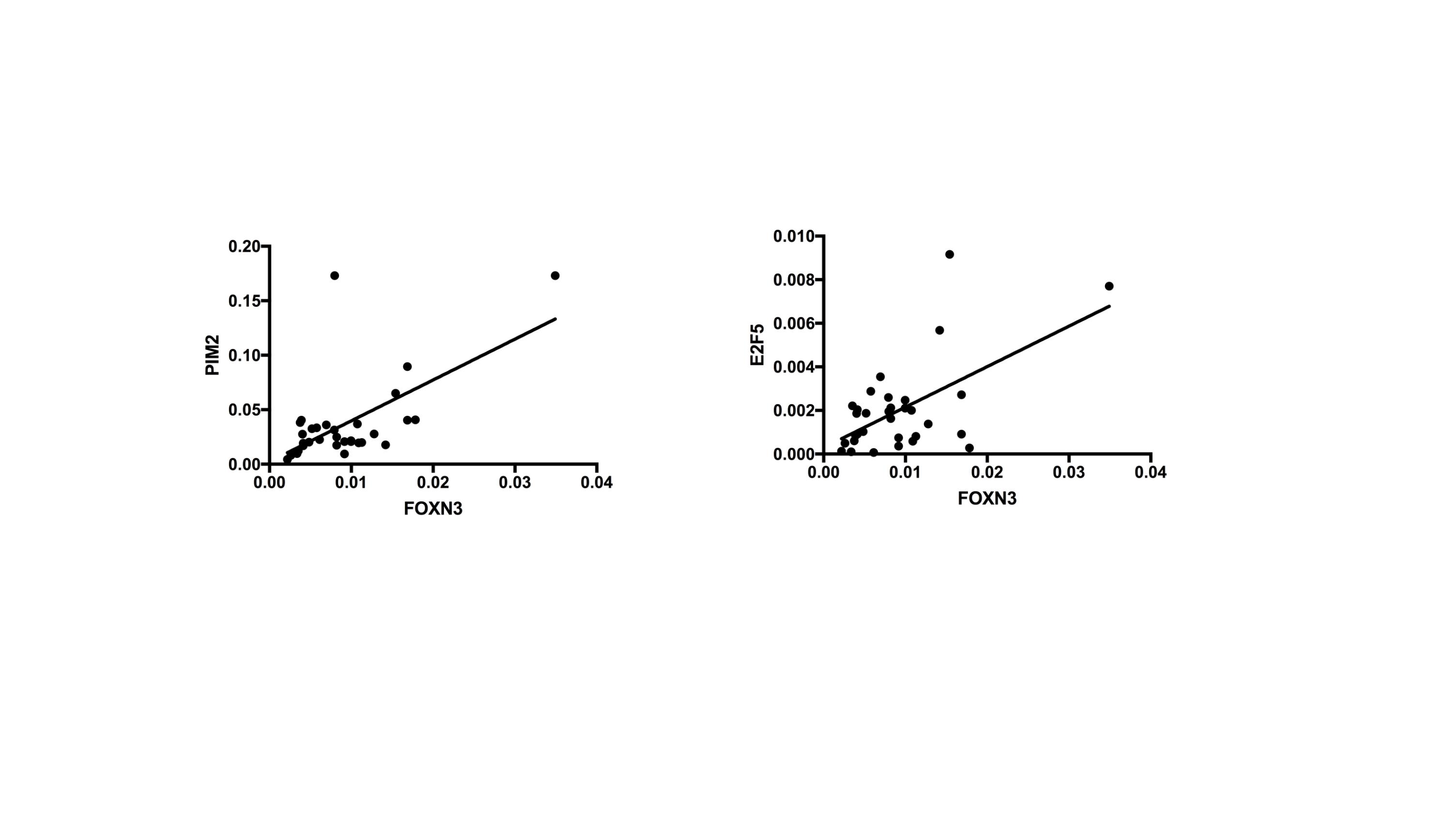


**A**

**B**

*P*=0.930

lower FOXN3 expression

higher FOXN3 expression

lower FOXN3 expression

higher FOXN3 expression

**100**

**100**

*P*=0.364

**50**

**0**

**50**

**0**

**0**

**50**

**100**

**150**

**0**

**2000**

**4000**

**6000**

**Time(month)**

**Time（day）**

**C**

*P*=0.319

lower FOXN3 expression

higher FOXN3 expression

**100**

**50**

**0**

**0**

**50**

**100**

**150**

**Time(month)**

Figure S3: Figure S3A, S3B The impact of FOXN3 on OS in AML patients from TCGA

AML database (A, n=136) and Beat AML database (B, n=392).

Figure S3C The impact of FOXN3 on RFS in AML patients from TCGA AML database

(n=136).


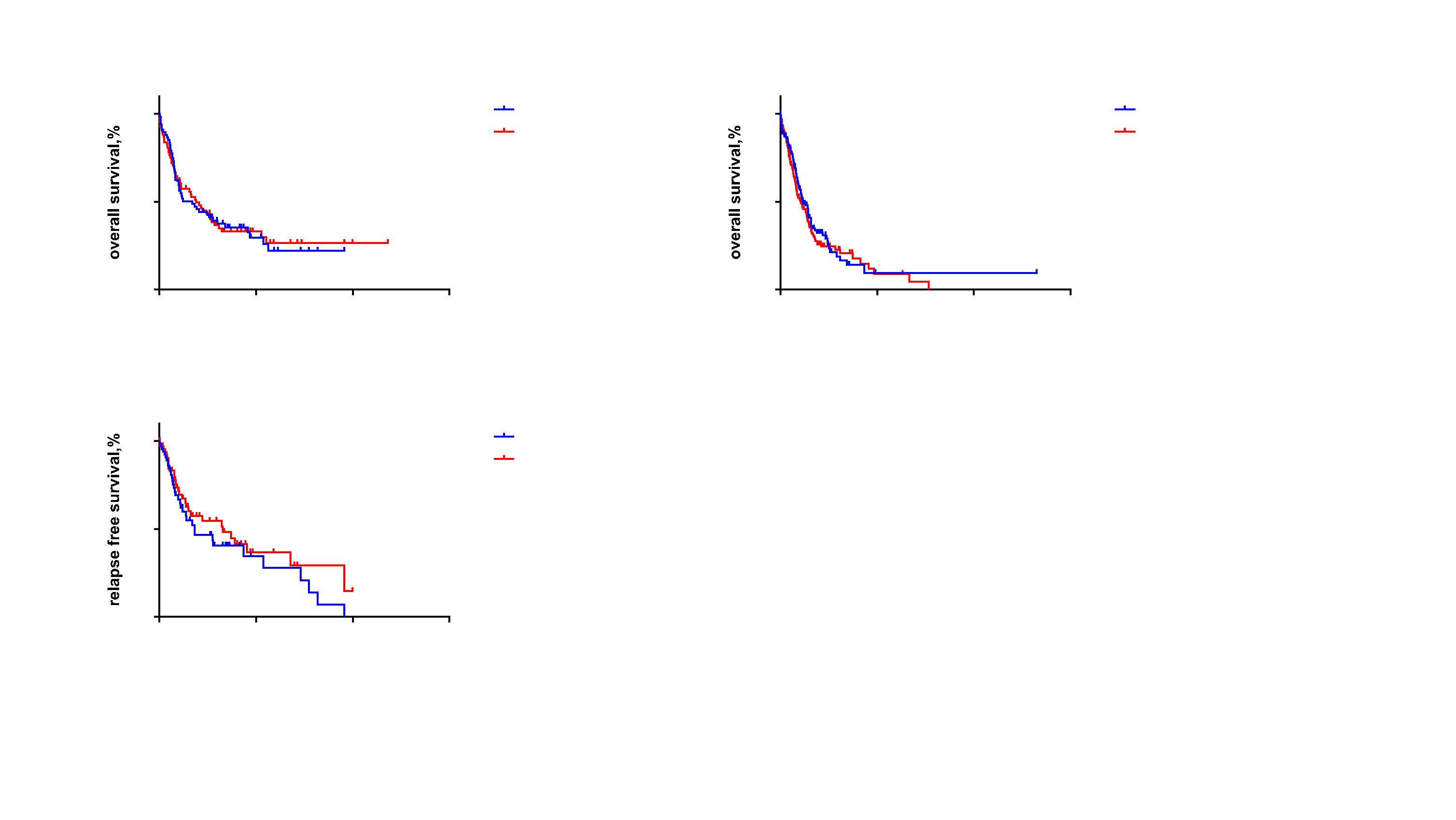


**A**

**B**

**200**

*P*=0.0005

lower FOXN3 expression

higher FOXN3 expression

**200**

*P*=0.050

lower FOXN3 expression

higher FOXN3 expression

**150**

**100**

**50**

**150**

**100**

**50**

***

**0**

**0**

**positive**

**negative**

**positive**

**negative**

**NPM1 gene mutation**

**DNMT3A gene mutation**

**D**

**C**

**200**

**200**

*P*=0.311

*P*=0.850

lower FOXN3 expression

higher FOXN3 expression

lower FOXN3 expression

higher FOXN3 expression

**150**

**100**

**50**

**150**

**100**

**50**

**0**

**0**

**positive**

**negative**

**positive**

**negative**

**ASXL1 gene mutation**

**TP53 gene mutation**

Figure S4:The association between lower FOXN3 expression with NPM1 gene mutation (A),

DNMT3A gene mutation (B), TP53 gene mutation (C) and ASXL1 gene mutation (D) from

Beat AML database (n=397).


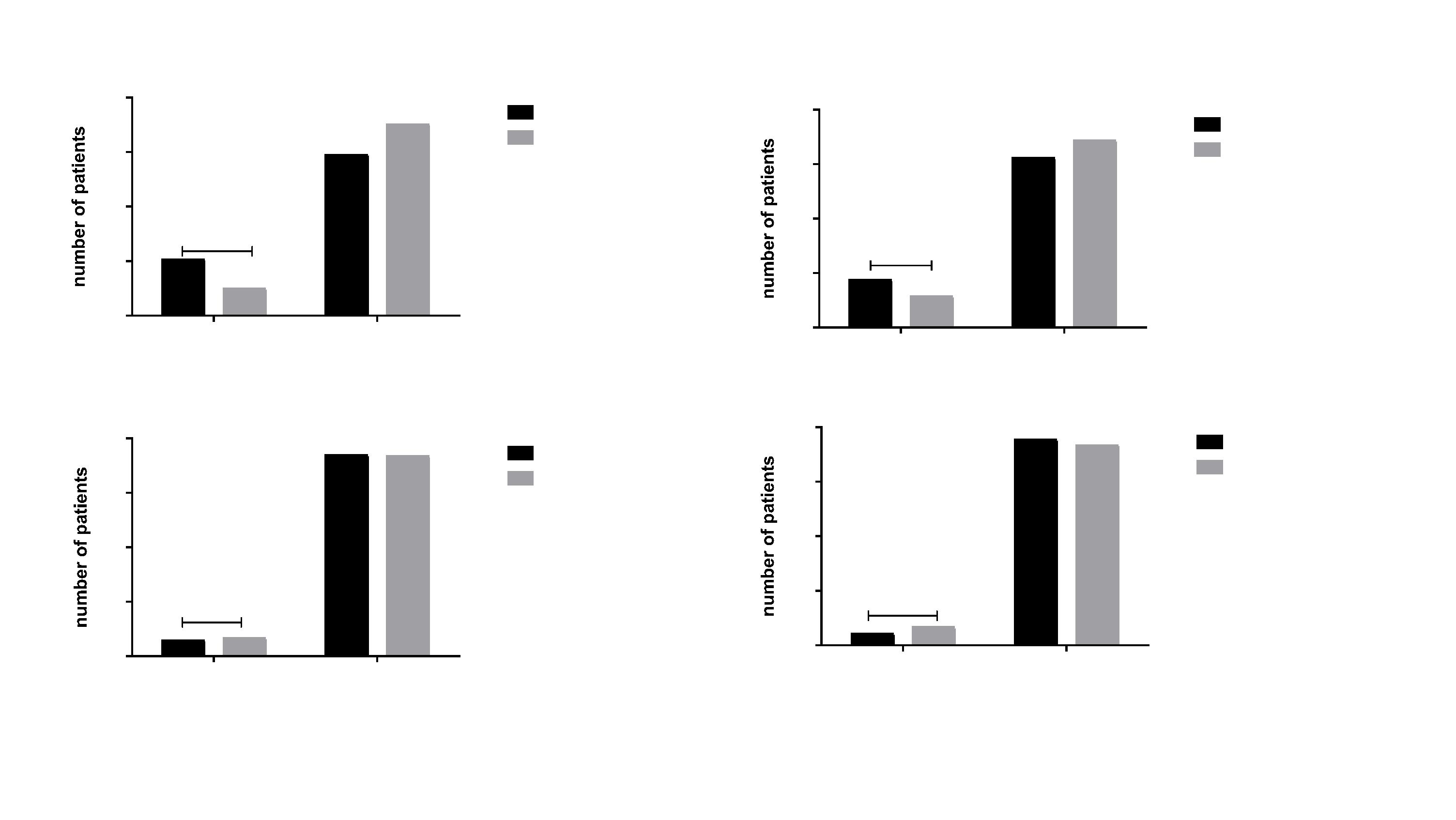

Supplement: Supplementary file 1 [file IJLH-42-270-s001.docx]
